# Emerging nanogenerators: Powering the Internet of Things by high entropy energy

**DOI:** 10.1016/j.isci.2021.102358

**Published:** 2021-04-26

**Authors:** Ya Yang, Zhong Lin Wang

**Affiliations:** 1 CAS Center for Excellence in Nanoscience, Beijing Key Laboratory of Micro-nano Energy and Sensor, Beijing Institute of Nanoenergy and Nanosystems, Chinese Academy of Sciences, Beijing, 101400, P. R. China; 2 School of Nanoscience and Technology, University of Chinese Academy of Sciences, Beijing, 100049, P. R. China; 3 School of Material Science and Engineering, Georgia Institute of Technology, Atlanta, GA 30332-0245, USA

**Figure:**
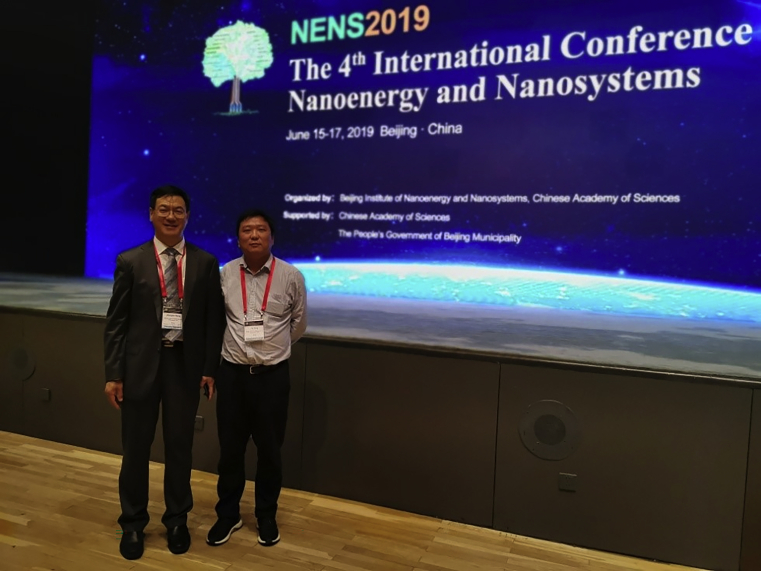
Prof. Zhong Lin Wang (left) and Prof. Ya Yang (right) in the fourth International Conference on Nanoenergy and Nanosystems in 2019.

The subject area of nanogenerators has today become a highly interdisciplinary field that deeply involves materials, information science, physics, chemistry, electronics, mechanics and even medical science.

Looking to the future of nanogenerators, one direction would be to develop new materials with different properties, as well as to investigate the mechanism of their action from the microscopic perspective.

There are a vast number of energy sources that are available for humans to choose from considering the future of energy. Some of them are obvious, such as fossil fuels, flowing water, the heat inside the earth and Sun. Some sources, though very common in our surroundings, are not that noticeable for energy harvesting, especially the mechanical energies at low frequencies, such as human motion, walking, raindrops, etc., which have been referred to as high entropy energy (Wang, 2019). *How can we harness this energy that is all around us, but not yet utilized for much of anything?* Harvesting that energy could provide a solution for everyday power needs and is becoming more and more important to consider for a sustainable energy for the “Internet of Things”, a network based on numerous smart devices everywhere. One of the most promising routes then is through nanogenerators. Nanogenerators are a field that utilize the Maxwell's displacement current to effectively convert mechanical energy into electric power/signal.

As a new type of energy harvester, nanogenerators operate on a small scale and have a huge potential to be utilized in various applications such as nanodevice/microdevice energy suppliers, self-powered sensor systems, blue energy, wireless power transmission, rain drip energy, wind energy, high voltage power sources, and in many other research fields. It is becoming an indispensable piece of the puzzle of sustainable energy.

Prof. Zhong Lin Wang and Prof. Ya Yang's research groups are engaged in extensive and exciting works of emerging nanogenerators and are field-leading scientists. In 2020, the two came together to lead a special issue at *iScience*, https://www.sciencedirect.com/journal/iscience/special-issue/10F5L60DXWN, covering a breadth of emerging nanogenerators such as piezoelectric, triboelectric, and hybridized nanogenerators from the fundamental materials to the applications of various nanogenerators such as soft robots, machines, medical mentoring, human-machine interfaces, and ocean wave energy scavenging. Here, we introduce you to Z.L.W. and Y.Y. as they introduce the field and provide their perspectives for the future. Through the publication of these papers in this special issue, they hope that it can push the field of nanogenerators forward toward high entropy energy scavenging.

## A subject built on interdisciplinarity

The subject area of nanogenerators has today become a highly interdisciplinary field that deeply involves materials, information science, physics, chemistry, electronics, mechanics, and even medical science. It is quickly expanding and attracting scientists and professionals from different communities, as can be indicated from the publication numbers on the subject. One particular reason for this is that there are very few limitations on the materials for nanogenerators, and many known and new materials could be used for this research. On the big picture side, we are confronting the energy crisis in a global context. There is a strong demand for finding new energy sources, especially sources that are “greener”. Further, nanogenerators harvest energy from the surroundings and can be assembled on a small scale and thus a great option as an energy supplier for portable or even nanodevices/microdevices. With such features, the nanogenerators could greatly speed up building the “Internet of Things”, which calls for decentralized and sustainable energy suppliers.

## A physics beginning

The story of nanogenerators starts from an additional term, now known as Wang term, in Maxwell's equations $\partial {\text{P}}_{\text{s}}/\partial \text{t}$, due to the non-electric field-induced and strain-related polarization, which is the driving force for converting mechanical energy into electricity (Wang, 2020). The prototype, a piezoelectric nanogenerator, was first reported in 2006 (Wang and Song, 2006) to scavenge mechanical energy by using the piezoelectric materials for powering light-emitting diodes, liquid crystal display, and so on. Another important member of the family, triboelectric nanogenerators, can harvest mechanical energy via contact electrification and electrostatic induction by using periodical contact and separation between two different materials, which was firstly demonstrated in 2012 (Fan et al., 2012).

## Frontiers of research

To scavenge multiple energies from environments, various hybridized and coupled nanogenerators have been explored (Xu et al., 2009). Electromagnetic-triboelectric hybridized nanogenerators were reported to simultaneously harvest one type of mechanical energy via two different effects and significantly enhance the energy conversion efficiency compared with the individual nanogenerators (Wang and Yang, 2017; Wu et al., 2015). The coupled nanogenerators, in which two or more different nanogenerators have the same electrodes but different energy harvesting capabilities (Zhang et al., 2017), have a smaller size, lower cost, and higher conversion efficiency than those simply integrating the different nanogenerators. Moreover, some new physical effects such as thermo-phototronic effect and ferro-pyro-phototronic effect have been uncovered in the coupled nanogenerators (Zhang and Yang, 2017; Zhao et al., 2018; Yang, 2021), which may have potential applications in solar cells, photodetectors, and so on.

## What's to come

Looking to the future of nanogenerators, one direction would be to develop new materials with different properties, as well as to investigate the mechanism of their action from the microscopic perspective. An equally important prospect is to apply nanogenerators to new energy sources. One example is harvesting energy from the surface waves of oceans. Some efforts with nanogenerators are ongoing (Wang, 2017), and it has huge potential to meet the energy need on a macro level. Integrating the nanogenerators into a system to realize a large energy output will be another research direction, where 10 or 100 W power sources may be achieved by assembling hundreds or thousands of nanogenerators for powering many electric devices with large power consumption. For the coupled nanogenerators, how to realize the coupling enhancement with higher energy conversion efficiency will be one of the important research directions, where the enhancement ratio may be dependent on the different materials, structures, and devices.

**Figure:**
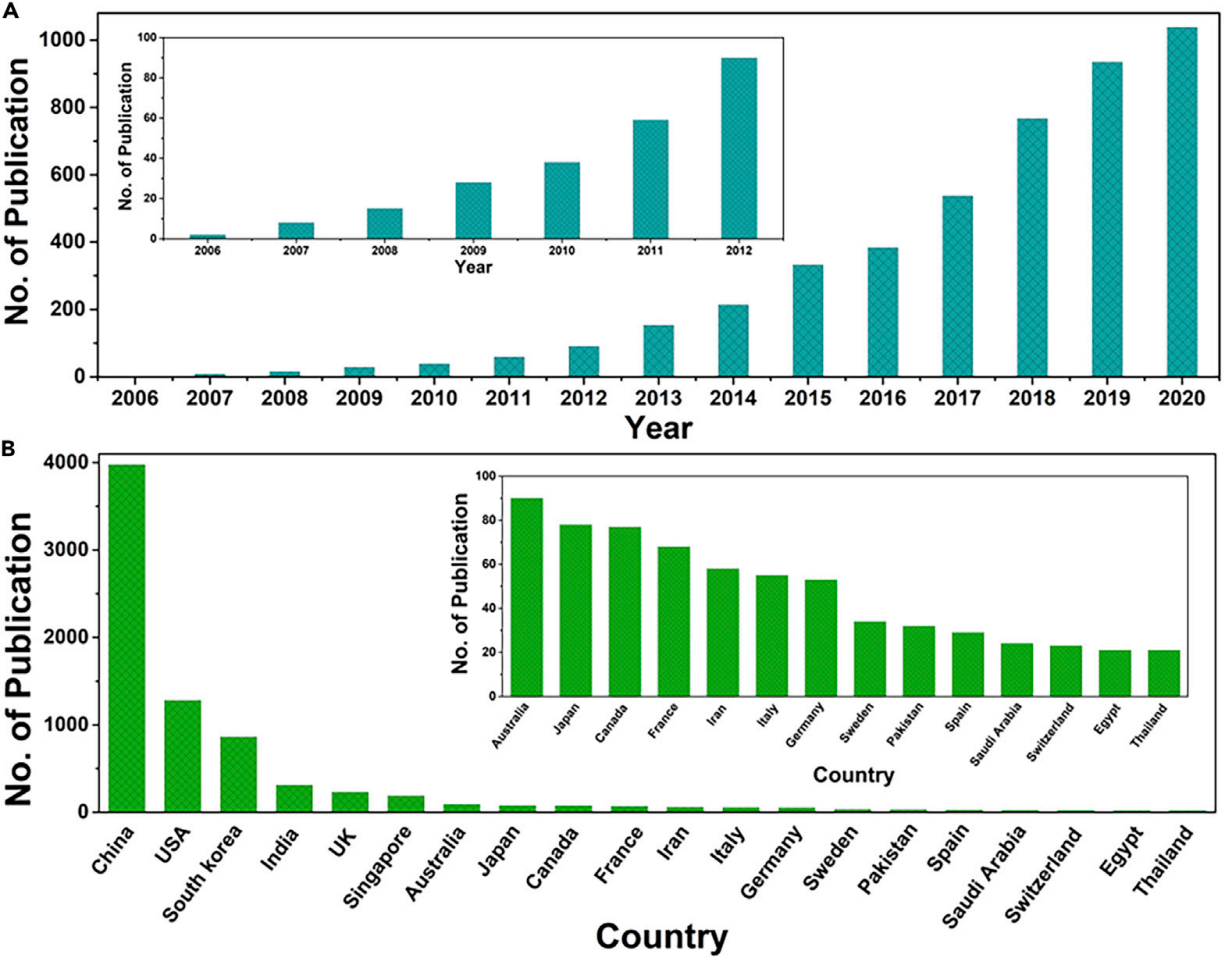
Statistics of publications from Web of Science, accessed March 2021. The number of publications in each year (A) and country (B) when "Nanogenerator" was used as the keyword for search in Web of Science.
